# The Longitudinal Impact of Maternal Depression and Neighborhood Social Context on Adolescent Mental Health

**DOI:** 10.3389/fped.2022.854418

**Published:** 2022-06-23

**Authors:** Daphne Lew, Hong Xian, Travis Loux, Enbal Shacham, Darcell Scharff

**Affiliations:** ^1^Division of Biostatistics, Washington University in St. Louis School of Medicine, Saint Louis, MO, United States; ^2^Center for Population Health Informatics, Institute for Informatics, Washington University in St. Louis School of Medicine, Saint Louis, MO, United States; ^3^Department of Epidemiology and Biostatistics, College for Public Health and Social Justice, Saint Louis University, Saint Louis, MO, United States; ^4^Department of Behavioral Science and Health Education, College for Public Health and Social Justice, Saint Louis University, Saint Louis, MO, United States

**Keywords:** mental health, neighborhoods, maternal-child health, longitudinal studies, depression, anxiety, collective efficacy

## Abstract

**Purpose:**

Maternal depression and neighborhood characteristics are known to be associated both with each other and with adolescent mental health outcomes. These exposures are also subject to change throughout the life of a child. This study sought to identify multi-trajectories of maternal depression (MD) and self-reported neighborhood collective efficacy (NCE) over a 12-year period and determine whether these trajectories are differentially associated with adolescent mental health.

**Methods:**

Data from the Fragile Families and Child Wellbeing study, a longitudinal cohort study of new parents and their children, were used. Maternal depression (MD) and self-reported NCE when the child was 3, 5, 9, and 15 years of age were the primary exposures of interest. Adolescent depression and anxiety symptomology when the child was 15 years of age were the primary outcomes. Primary analyses were conducted using multi-trajectory modeling and linear regressions.

**Results:**

Five multi-trajectories were identified, two of which were characterized by no MD but either high or low NCE, and three of which were characterized by similarly moderate levels of NCE but either increasing, decreasing, or consistently high MD. Children of mothers with increasing or consistently high depressive symptomology and moderate NCE had significantly higher depression and anxiety scores compared to children of mothers with no depressive symptomology and high NCE.

**Conclusion:**

Adolescents with consistent and proximal exposure to MD are most likely to suffer from adverse mental health and should be provided with appropriate support systems to mitigate these outcomes.

## Introduction

Nearly half of all American adolescents aged 13–18 have experienced some lifetime mental illness, and nearly 20% of adolescents experience comorbid mental disorders (1). The prevalence of depression among American adolescents aged 12–17 years is estimated to be 6%, while the rate of adolescents experiencing any major depressive episode in the past year is estimated to be as high as 12.5% (2, 3). Nearly one-third of American adolescents (31.9%) are estimated to experience any lifetime anxiety disorder, making it the most common mental health condition among this population (1). Mental illness in adolescence is known to co-occur with substance use (4), such that individuals experiencing mental illness are much more likely to use substances than teens without mental illness. Moreover, rates of major depression and depressive episodes in this population appear to be increasing over the past decade (3). Among adolescents who suffered from major depressive disorder, one study identified a fivefold increase in the risk of suicide attempts in adulthood and more than twice the risk of developing major depressive disorder in adulthood, compared to adolescents who did not experience mental illness (5). Findings with regards to anxiety disorders during adolescence are similar, indicating that the vast majority of children and adolescents who experience any anxiety disorder will continue to experience anxiety disorders or other mental health conditions throughout the course of their lives (6). Given the strong relationships between physical and mental health among adults, this increasing burden of mental illness among American adolescents is concerning.

There are many well-defined risk and protective factors associated with adolescent mental health. These risk factors range from intrapersonal factors such as genetic predisposition (7), to societal factors such as community violence exposure (8) or poor physical environment quality (9). Two risk factors that are known to be strongly associated with adolescent mental health outcomes are the mental health of the child’s mother and the child’s neighborhood environment.

The relationship between maternal mental illness and adverse mental health outcomes for a mother’s child is fairly robust. Research has shown that exposure to maternal mental illness during the immediate postpartum period (10), as well as during later periods of the child’s life (11) is significantly associated with development of psychopathology in the child. It has also been shown that the earlier the child is first exposed to maternal mental illness and the more consistent and severe the exposure is, the greater the risk for development of mental illness in childhood and beyond (12, 13). Further, the relationship between maternal and child mental health is consistent with regards to different types of psychopathology development (14, 15) and different populations of mothers (16).

With regards to the neighborhood environment, both physical and social components of the neighborhood environments are known to be associated with adverse physical and mental health outcomes of children (17, 18). Physical components include such characteristics as the built environment, crowding, noise or traffic levels, presence of parks, and presence of food, tobacco, or alcohol outlets. On the other hand, social components are characteristics of the neighborhood related to overall levels of social cohesion, social support, social capitol, and social control. Expectedly, adverse physical components are associated with poorer mental health outcomes of children (9, 19), whereas positive social components are associated with more favorable mental health outcomes (20, 21). Interestingly, research has shown that often the social components of an individual’s neighborhood can buffer, mediate, or confound the effect of adverse physical components on mental health outcomes (20–23).

While much work has been done to indicate the strong impact of maternal mental health and neighborhood environments on adolescent mental health outcomes, it is important to note that both of these risk factors are subject to change over time. Moreover, the relationship between maternal mental health and neighborhood environments is highly intertwined, with research indicating robust relationships between these two factors in and of themselves (22, 24, 25). Given the fact that these factors are not static and may be highly correlated, examining simultaneous changes in these risk factors over time and the impact that these changes may have on adolescent mental health outcomes is warranted.

As such, the aim of the present analysis is to determine whether simultaneous trajectories of maternal mental health and neighborhood social environments exist in a longitudinal cohort of mothers. Further, we would like to determine whether any identified trajectories have differential impacts on the mental health of the mother’s child. It is hypothesized that trajectories with high levels of adverse maternal mental health would be associated with worse mental health outcomes for the child, but that children whose mothers report more favorable neighborhood social environments would fare better than those children whose mothers report less favorable neighborhood social environments.

## Materials and Methods

### Participants

Data used in the present analyses were from participants in the Fragile Families and Child Wellbeing Study (FFCWS), which was a longitudinal cohort study of births from primarily non-marital families (births from mothers who were unmarried) (26). Specific details about the study, sampling procedures, and exclusion criteria have been described elsewhere (26). In short, the baseline sample included mothers who gave birth between 1998 and 2000 and was comprised of approximately 3,600 non-marital births and 1,100 marital births. The mothers were sampled from 75 hospitals within 20 large cities in the United States.

**FIGURE 1:**
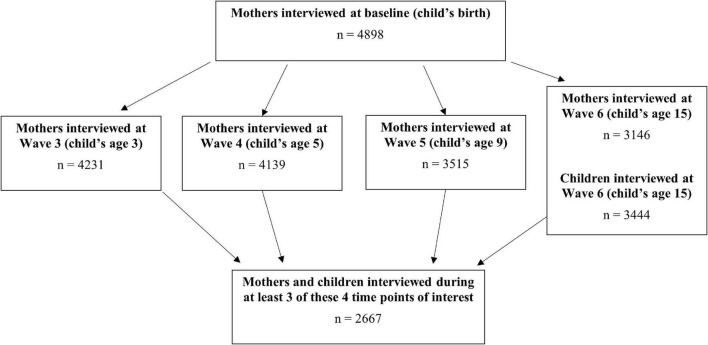
Flow diagram depicting analytic sample of interest.

The cohort of families recruited into the FFCWS was interviewed at baseline, shortly after the child’s birth, and then again when the child was approximately 1, 3, 5, 9, and 15 years of age. For the present analysis, only data from when the child was 3, 5, 9, and 15 years of age was used, as all measures of interest were assessed consistently during these time points. Mothers and their children who were interviewed during at least three of these four points in time were included in the analytic sample (Figure 1). Given the publicly available and de-identified nature of these data, this study was exempt from IRB review.

### Measures

A listing of all items and response options included in the measures of interest can be found in Supplementary Tables 1–3. Descriptions of these measures are included below.

#### Maternal Mental Health

Maternal mental health was measured as depressive symptomology using the Composite International Diagnostic Interview—Short Form (CIDI-SF) scale (27). Though cutoffs indicating likely cases of depression were available in the data, the overall CIDI-SF score (ranging from 0 to 8) was used to characterize maternal depression (MD; Cronbach’s α ranging from 0.77 to 0.87 across waves). Using this score, as opposed to the dichotomous cutoff, provided a greater degree of variability in maternal depressive symptomology and resulted in better model fit.

#### Neighborhood Social Environment

The neighborhood social environment was measured by a scale representing neighborhood collective efficacy (NCE). NCE has been shown to be highly representative of the overall social context that an individual experiences in their neighborhood (28). This construct was measured using a nine-item scale derived from existing measures similar to those used in the Project on Human Development in Chicago Neighborhoods (29). The overall scale (range 9–36) was created by summing the nine items of interest and then reverse-scoring such that higher scores corresponded to higher levels of NCE (α ranging from 0.84 to 0.87 across waves).

#### Adolescent Mental Health

Two measures of adolescent mental health were used as the primary outcomes. The first measure was a five-item subscale of the Center for Epidemiologic Studies Depression Scale (CES-D) (30), which was used to measure depressive symptomology (α = 0.75). The specific subscale of the CES-D used herein has been validated by prior studies and has been shown to be particularly effective among racially and ethnically diverse populations (31). The second measure was the six-item anxiety subscale from the Brief Symptom Inventory 18 (BSI 18) (32), which was used to measure adolescent anxiety symptomology (α = 0.76). Both of these scales were treated as continuous variables and reverse-scored such that higher scores corresponded to higher levels of symptomology. These measures were assessed at the final time point, when the child was 15 years of age.

#### Covariates

Several important covariates were taken from the baseline time point of interest, when the child was 3 years of age. Measures of the mother’s substance use were dichotomous indicators of whether the mother smoked cigarettes, drank alcohol, or used illicit drugs (including marijuana) at any time in the previous month. A dichotomous measure of violence exposure was also used. Mothers that indicated they had seen “someone get hit, slapped, punched, or beaten up,” “someone else get attacked with a weapon,” or “someone get shot at by someone else” at least once in the past year were categorized as having violence exposure. Household income was reflected by a dichotomous variable indicating whether the family’s household income was below the federal poverty level. A dichotomous variable indicating whether families experienced any material hardship in the past year was used to reflect income instability in the home. Finally, whether or not the family had moved since the last interview wave was included as a measure of housing instability during the time period.

Measures of the child’s substance use during adolescence, taken when the child was 15 years old, were also included. Similar to the maternal substance use variables, these were three dichotomous variables indicating whether the child smoked cigarettes, drank alcohol, or used illicit drugs in the past 30 days.

#### Demographic Characteristics

Sociodemographic covariates were taken from the baseline time point of interest (when the child was age 3). These covariates included maternal education level (less than high school/high school graduate or equivalent/some college/college graduate or higher), the mother’s relationship with the child’s father (married/not married but living together/not living together but romantically involved/separated, divorced, or widowed/friend/no relationship), the mother’s age, the child’s biological sex, and the mother’s race/ethnicity (non-hispanic Black/non-hispanic White/Hispanic/Other).

### Statistical Analysis

Descriptive statistics of all variables were calculated. Frequencies and percentages were calculated for categorical variables. For continuous variables, means, and standard deviations (SD) were calculated when the data were approximately normally distributed, whereas medians and inter-quartile ranges (IQR) were calculated when the data were skewed.

In order to identify simultaneous patterns of MD and NCE over time, multi-trajectory modeling (MTM) was used (33). This method is an extension of group-based trajectory modeling (34) that allows for the identification of latent groups in a population following similar patterns of multiple variables over time, as opposed to just one. In this way, MTM uses information from both variables of interest to identify one set of trajectories that characterize patterns of these variables over time. This analysis was performed using the TRAJ procedure (35) in SAS 9.4 (SAS Institute, Cary, NC). Model selection was conducted using fit statistics, such as the Bayesian information criteria (BIC), and theoretical interpretation of the groups. The underlying distributions associated with the variables of interest were the zero-inflated Poisson distribution for the CIDI-SF scale score and the censored normal distribution for the NCE score.

Mothers were classified into one of the multi-trajectory groups (MTGs) based on their highest probability of group membership. Then, the socio-demographic characteristics and covariates of mothers and their children were compared across MTGs using chi-square or Fisher’s exact tests for categorical variables and ANOVA for continuous variables. Finally, two separate ordinary least squares linear regressions were used to examine the relationship between the mother’s MTG membership and either adolescent depression or anxiety. Four blocks of covariates were used, such that five model estimates were generated as follows: (1) crude model with no covariates; (2) demographic covariates only (child’s sex, mother’s age, mother’s race, mother’s education level, and mother’s relationship with the child’s father); (3) demographic covariates and maternal substance use variables (cigarette use, alcohol use, and illicit drug use); (4) demographic covariates, maternal substance use variables, and risk factors (violence exposure, material hardship, income to poverty ratio, whether the family has moved); (5) demographic covariates, maternal substance use variables, risk factors, and child substance use variables (cigarette use, alcohol use, and illicit drug use). All analyses were performed in SAS for Windows version 9.4 (SAS Institute, Cary, NC) and an α of 0.05 was used to determine statistical significance.

## Results

### Sample Characteristics

A total of 2,667 mothers and their children were included in the analytic sample (Figure 1). The median age of mothers when the child was 3 years old was 27 years (IQR: 23, 32), 50.7% of mothers were non-Hispanic black, and 23.6% of mothers had less than a high school education (Table 1). Around one-third of mothers were married to the child’s father (33.4%) and another 18.6% of mothers were living with the father. The distribution of male and female children was roughly similar (51.2 and 48.8%, respectively). Descriptive statistics of all exposures, covariates, and outcomes of interest are shown in Table 1.

**TABLE 1:** Descriptive statistics of all demographic characteristics and covariates.

Characteristic	N (%), median (IQR), or mean ± SD
**Demographic (Reported at baseline, child’s age 3)**	
Child’s biological sex	
*Male*	1,365 (51.2)
*Female*	1,302 (48.8)
Mother’s race	
*Non-hispanic Black*	1,350 (50.7)
*Non-hispanic White*	593 (22.3)
*Hispanic*	622 (23.4)
*Other*	96 (3.6)
Mother’s age	27 (23, 32)
Highest level of education	
*Less than high school*	628 (23.6)
*High school or equivalent*	772 (29.0)
*Some college or technical school*	888 (33.3)
*College degree or higher*	378 (14.2)
Relationship with child’s father	
*Married to father*	889 (33.4)
*Not married but living with father*	495 (18.6)
*Not living with father but romantically involved*	157 (5.9)
*Separated, divorced, or widowed*	171 (6.4)
*Friends with father*	453 (17.0)
*No relationship with father*	498 (18.7)
**Maternal mental health (Reported at baseline, child’s age 3)**	
Any depression symptomology	564 (21.2)
Depression score among mothers with any symptomology	5.7 ± 1.4
Past month alcohol use	1,383 (52.1)
Past month cigarette use	504 (22.3)
Past month illicit substance use	196 (7.4)
Any violence exposure in the past year	816 (36.2)
**Home and neighborhood environment (Reported at baseline, child’s age 3)**	
Neighborhood collective efficacy score	17.4 ± 6.4
Dichotomized low neighborhood collective efficacy	829 (38.1)
Household income at or below poverty level	1,044 (39.2)
Any material hardship in past year	1,196 (45.0)
Moved homes since last interview	1,244 (46.7)
**Adolescent health (Reported at final time point, child’s age 15)**	
Adolescent depression score	2 (0, 5)
Adolescent anxiety score	4 (2, 7.5)
Past month alcohol use	111 (4.3)
Past month cigarette use	40 (1.6)
Past month illicit drug use	189 (8.1)

Some differences in the demographic characteristics of mothers included in the analytic sample vs. those excluded from the sample were observed. A higher proportion of mothers in the excluded sample were Hispanic, had less than a high school education, endorsed cigarette use, had moved homes since the last interview wave, and reported household incomes at or below the poverty level (Supplementary Table 2). However, no significant differences in the mother’s depression scores or neighborhood collective efficacy at baseline, or in the child’s depression or anxiety scores in adolescence were observed when comparing the individuals included in the analytic sample to those excluded.

### Multi-Trajectory Modeling

Model fit statistics for all assessed MTM models are shown in Supplementary Table 3. Based on these model fit statistics, the five-trajectory model was preferable as it had the smallest BIC. Further, the theoretical interpretation of the five identified multi-trajectories was straightforward.

**FIGURE 2:**
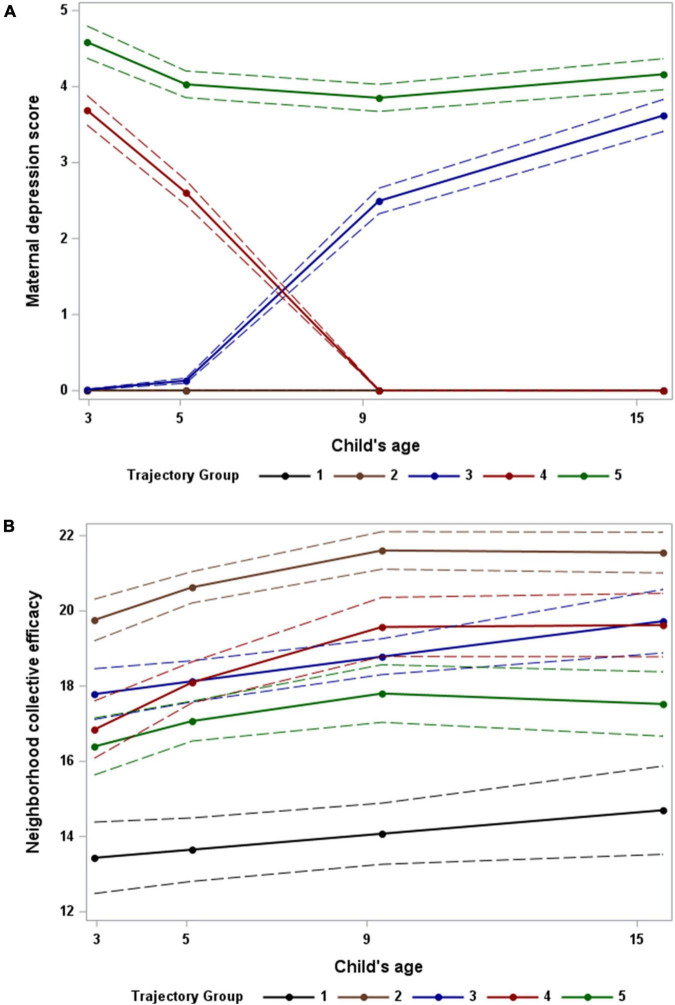
Multi-trajectories of maternal depression **(A)** and maternal reported neighborhood collective efficacy **(B)** during the period from when the child is 3 to 15 years of age. Dashed lines reflect 95% confidence intervals for model predictions. Note: Trajectories 1 and 2 in panel **(A)** both have depression scores of zero at all time points.

Figure 2 shows the mean MD (A) and NCE (B) scores of mothers in each of the five trajectory groups across all time points. In Figure 2A, MTGs 1 and 2 are both characterized by mothers with no depressive symptomology throughout the time period. When examining these two trajectory groups in Figure 2B, it can be seen that mothers in MTG 1 generally had much lower NCE than mothers in MTG 2. In contrast, MTGs 3, 4, and 5 shown in Figure 2A all have very distinct patterns of increasing, decreasing, and consistently high maternal depressive symptomology, respectively. When examining these three trajectory groups in Figure 2B, however, it is clear that the levels of neighborhood collective efficacy among mothers in these groups were all fairly similar. Based on these observed trends, the five trajectory groups were named as follows: (1) Low MD, low NCE (*n* = 312); (2) Low MD, high NCE (*n* = 1,232); (3) Increasing MD, moderate NCE (*n* = 347); (4) Decreasing MD, moderate NCE (*n* = 396); (5) High MD, moderate NCE (*n* = 380).

### Demographic Characteristics Across Multi-Trajectory Groups

When examining distributions of sociodemographic characteristics across MTGs, significant differences were observed for all variables other than the child’s biological sex (Table 2). MTG 2 was expected to be the lowest risk group, as this trajectory was characterized by mothers with no depressive symptomology and high NCE. MTG 1 looks to be very similar to MTG 2, except in the levels of NCE. However, upon examining the sociodemographic characteristics of these two groups, meaningful differences were observed. In particular, MTG 2 vs. 1 was comprised of mothers who were older (mean age: 29.0 years vs. 28.0 years, respectively), a higher proportion of non-hispanic white mothers (24.7% vs. 9.3%, respectively), a higher proportion of mothers with a college degree or higher (19.2% vs. 6.4%, respectively), and a higher proportion of mothers that were married to the child’s father (40% vs. 21.9%, respectively). MTG 2, compared to 1, also had a lower proportion of mothers experiencing violence exposure (27.4% vs. 43.8%, respectively), material hardship (34.7% vs. 43.1%, respectively), and poverty (32.9% vs. 54.2%, respectively). All of these pairwise differences between MTG 1 and 2 were statistically significant (*p* < 0.05). When examining the other groups, it was found that MTG 5 had the highest proportions of mothers endorsing substance use (35.9% for cigarette use, 59.4% for alcohol use, and 15.8% for illicit drug use), as well as the highest proportion of mothers reporting any material hardship (67.8%). MTG 3 had the youngest group of mothers (mean age: 27.5 years), compared to all other groups.

**TABLE 2:** Baseline (child’s age 3) sociodemographic and risk characteristics of mothers and children in each trajectory group.

Variable label	Variable value	(1) Low MD, low NCE (*n* = 312)	(2) Low MD, high NCE (*n* = 1,232)	(3) Increasing MD, moderate NCE (n = 347)	(4) Decreasing MD, moderate NCE (n = 396)	(5) High MD, moderate NCE (n = 380)	*p*-value
Child’s biological sex	Male	156 (50)	624 (50.65)	191 (55.04)	190 (47.98)	204 (53.68)	0.291
	Female	156 (50)	608 (49.35)	156 (44.96)	206 (52.02)	176 (46.32)	
Mother’s age when the child is 3 years old		27.98 ± 5.82 (311)	28.96 ± 6.2 (1,232)	27.52 ± 5.83 (347)	27.61 ± 5.81 (396)	27.73 ± 6.11 (380)	<0.001
Mother’s race	White, non-hispanic	29 (9.29)	304 (24.74)	85 (24.57)	87 (21.97)	88 (23.28)	<0.001
	Black, non-hispanic	184 (58.97)	582 (47.36)	172 (49.71)	201 (50.76)	211 (55.82)	
	Hispanic	87 (27.88)	296 (24.08)	79 (22.83)	90 (22.73)	70 (18.52)	
	Other	12 (3.85)	47 (3.82)	10 (2.89)	18 (4.55)	9 (2.38)	
Mother’s education when the child is 3 years old	Less than high school	100 (32.05)	243 (19.72)	91 (26.22)	86 (21.72)	108 (28.5)	<0.001
	High school or equivalent	94 (30.13)	350 (28.41)	98 (28.24)	112 (28.28)	118 (31.13)	
	Some college or technical school	98 (31.41)	403 (32.71)	109 (31.41)	150 (37.88)	128 (33.77)	
	College degree or higher	20 (6.41)	236 (19.16)	49 (14.12)	48 (12.12)	25 (6.6)	
Mother’s relationship with the father when the child is 3 years old	Married to father	68 (21.94)	492 (40)	117 (33.72)	117 (29.55)	95 (25)	<0.001
	Not married but living with father	65 (20.97)	226 (18.37)	57 (16.43)	79 (19.95)	68 (17.89)	
	Not living with father but romantically involved	28 (9.03)	61 (4.96)	26 (7.49)	20 (5.05)	22 (5.79)	
	Separated, divorced, or widowed	17 (5.48)	74 (6.02)	14 (4.03)	34 (8.59)	32 (8.42)	
	Friends with father	54 (17.42)	204 (16.59)	54 (15.56)	73 (18.43)	68 (17.89)	
	No relationship with father	78 (25.16)	173 (14.07)	79 (22.77)	73 (18.43)	95 (25)	
Maternal cigarette use	No	221 (82.46)	846 (83.27)	219 (73)	253 (75.07)	218 (64.12)	<0.001
	Yes	47 (17.54)	170 (16.73)	81 (27)	84 (24.93)	122 (35.88)	
Maternal alcohol use	No	175 (56.27)	598 (48.74)	175 (50.43)	172 (43.77)	154 (40.63)	<0.001
	Yes	136 (43.73)	629 (51.26)	172 (49.57)	221 (56.23)	225 (59.37)	
Maternal illicit drug use	No	299 (95.83)	1,180 (95.93)	320 (92.22)	349 (88.35)	320 (84.21)	<0.001
	Yes	13 (4.17)	50 (4.07)	27 (7.78)	46 (11.65)	60 (15.79)	
Any violence exposure	No	150 (56.18)	735 (72.56)	193 (64.33)	194 (57.91)	166 (48.97)	<0.001
	Yes	117 (43.82)	278 (27.44)	107 (35.67)	141 (42.09)	173 (51.03)	
Any material hardship	No	177 (56.91)	801 (65.33)	194 (55.91)	167 (42.39)	122 (32.19)	<0.001
	Yes	134 (43.09)	425 (34.67)	153 (44.09)	227 (57.61)	257 (67.81)	
Household income at or below poverty level	No	143 (45.83)	827 (67.13)	218 (62.82)	242 (61.11)	193 (50.79)	<0.001
	Yes	169 (54.17)	405 (32.87)	129 (37.18)	154 (38.89)	187 (49.21)	
Moved homes since last interview	No	154 (49.36)	707 (57.43)	179 (51.59)	198 (50)	184 (48.42)	0.003
	Yes	158 (50.64)	524 (42.57)	168 (48.41)	198 (50)	196 (51.58)	

### Relationship Between Multi-Trajectory Groups and Adolescent Mental Health

MTG 2, comprised of mothers with no depressive symptomology and high NCE, was used as the common reference for all regression models. In the crude models, children whose mothers were in MTG 3 had a depression score 0.502 points higher (95% CI: [0.135, 0.868]) and an anxiety score 0.608 points higher (95% CI: [0.129, 1.086]) than children whose mothers were in MTG 2 (Table 3). Similarly, children with mothers in MTG 5 had a depression score 0.734 points higher (95% CI: [0.385, 1.083]) and an anxiety score 1.142 points higher (95% CI: [0.683, 1.601]) than children with mothers in MTG 2 in the crude models. After controlling for covariates in blocks 1, 2, and 3 the significant association between mother’s membership in MTGs 3 and 5 and adverse adolescent mental health outcomes persisted (Table 3). Once adolescent substance use was controlled for in block 4, the relationship between mother’s membership in MTG 5 and adolescent depression symptomology was no longer significant, though the relationship with anxiety remained significant. In contrast, children whose mothers were classified into MTG 3 still had significantly higher depression scores after controlling for substance use but did not have higher anxiety symptomology.

**TABLE 3:** Results from adjusted models using trajectory group to predict adolescent mental health outcomes.

Outcome	Trajectory group	Crude parameter estimate [95% CI]	Crude *p*-value	Block 1^†^ parameter estimate [95% CI]	Block 1 *p*-value	Block 2^‡^ parameter estimate [95% CI]	Block 2 *p*-value	Block 3^⸸^ parameter estimate [95% CI]	Block 3 *p*-value	Block 4^+^ parameter estimate [95% CI]	Block 4 *p*-value
Adolescent depression score	(2) Low MD*, high NCE**	Reference		Reference		Reference		Reference		Reference	
	(1) Low MD, low NCE	0.206 [−0.175, 0.587]	0.2884	0.025 [−0.357, 0.407]	0.8982	0.12 [−0.291, 0.532]	0.5672	0.059 [−0.356, 0.473]	0.7814	0.11 [−0.302, 0.522]	0.6001
	(3) Increasing MD, moderate NCE	**0.502 [0.135, 0.868]**	**0.0073**	**0.479 [0.115, 0.843]**	**0.0100**	**0.539 [0.149, 0.929]**	**0.0068**	**0.511 [0.12, 0.902]**	**0.0105**	**0.573 [0.174, 0.971]**	**0.0049**
	(4) Decreasing MD, moderate NCE	0.31 [−0.033, 0.653]	0.0764	0.244 [−0.096, 0.584]	0.1590	0.347 [−0.025, 0.718]	0.0673	0.258 [−0.12, 0.636]	0.1805	0.308 [−0.074, 0.69]	0.1139
	(5) High MD, moderate NCE	**0.734 [0.385, 1.083]**	**<0.0001**	**0.633 [0.284, 0.982]**	**0.0004**	**0.613 [0.236, 0.99]**	**0.0014**	**0.462 [0.072, 0.851]**	**0.0203**	0.307 [−0.092, 0.707]	0.1318
Adolescent anxiety score	(2) Low MD, high NCE	Reference		Reference		Reference		Reference		Reference	
	(1) Low MD, low NCE	0.205 [−0.293, 0.703]	0.4199	0.104 [−0.402, 0.61]	0.6867	0.175 [−0.371, 0.721]	0.5300	0.122 [−0.428, 0.671]	0.6637	0.128 [−0.432, 0.689]	0.6531
	(3) Increasing MD, moderate NCE	**0.608 [0.129, 1.086]**	**0.0128**	**0.593 [0.113, 1.074]**	**0.0155**	**0.6 [0.083, 1.116]**	**0.0229**	**0.567 [0.05, 1.084]**	**0.0316**	0.517 [−0.021, 1.056]	0.0598
	(4) Decreasing MD, moderate NCE	0.268 [−0.182, 0.718]	0.2428	0.204 [−0.247, 0.654]	0.3751	0.252 [−0.242, 0.745]	0.3172	0.158 [−0.344, 0.66]	0.5377	0.165 [−0.356, 0.686]	0.5343
	(5) High MD, moderate NCE	**1.142 [0.683, 1.601]**	**<0.0001**	**1.091 [0.626, 1.556]**	**<0.0001**	**1.04 [0.537, 1.543]**	**<0.0001**	**0.858 [0.338, 1.378]**	**0.0012**	**0.822 [0.276, 1.368]**	**0.0032**

*Bolded estimates are statistically significant.*

*MD, maternal depression; ^**^NCE, neighborhood collective efficacy.

*^†^Block 1 covariates: child’s sex, mother’s age, mother’s race, mother’s education level, and mother’s relationship with child’s father.*

*^‡^Block 2 covariates: all block 1 variables plus mother’s cigarette use, alcohol use, and illicit drug use when the child is age 3.*

*^⸸^ Block 3 covariates: all block 2 variables plus violence exposure, material hardship, income to poverty ratio, and whether the family has moved in the past year when the child is age 3.*

*^+^Block 4 covariates: all block 3 variables plus adolescent cigarette, alcohol, and illicit drug use in the past month.*

## Discussion

The simultaneous patterns of MD and maternal-reported NCE identified in the present analysis provide important insight into how these two interrelated variables jointly change over time. Analyzing both variables simultaneously allowed for the identification of two groups of mothers with no depressive symptomology but very differently levels of NCE and three groups of mothers with similar levels of NCE but very different depression patterns. Had each of these variables been analyzed individually, the unique characteristics distinguishing these groups would likely have been overlooked. Upon examining the relationship between the MTGs and adolescent mental health outcomes, children with mothers that had increasing or persistently high depressive symptomology had significantly worse mental health in adolescence.

Little has been done to classify trajectories of neighborhood social characteristics over time, but patterns of depressive symptomology identified herein are consistent with existing research. For example, the groups of mothers with persistently high or low symptomology are similar to what has been shown in other trajectory analyses examining maternal mental health (11, 14, 36). These studies have also identified groups of mothers with increasing or decreasing symptomology throughout the time period, similar to MTGs 3 and 4 (14, 36). Interestingly, the groups identified in existing studies typically experienced at least moderate levels of symptomology throughout the time period, despite displaying consistent increases or decreases. In contrast, the current study found that mothers in these increasing or decreasing groups had little to no symptomology at the start or end of the time period, respectively.

The characteristics of mothers in the identified MTGs are also consistent with expectations based on previous literature. MTG 2, which was the lowest risk group of mothers exhibiting no MD and high NCE, was comprised of higher proportions of mothers that are typically thought to have greater societal advantages. For example, a larger proportion of mothers in this group were of white race, had at least some college education, and were married to the child’s father, and this group had the smallest proportion of mothers with violence exposure and material hardship. Other studies have shown that mothers with similarly favorable characteristics are also more likely to have positive perceptions of their neighborhood (37, 38). MTG 5, which was characterized by persistently high depressive symptomology and moderate NCE, had the highest proportions of mothers endorsing any cigarette use and fairly high proportions of mothers reporting material hardship or low household income. This is consistent with existing literature indicating that mothers suffering from depression are more likely to engage in substance use or experience substance use disorders (39, 40) and that low income levels are highly correlated with depressive symptomology among mothers (41).

In this study, children whose mothers had consistently high or increasing depressive symptomology throughout the time period had the worst mental health outcomes. This finding is expected based on existing literature indicating that more consistent and proximal exposures to adverse maternal mental health have the strongest effects on the mental health of the mother’s child (13). Consistent with literature indicating that positive social characteristics of a neighborhood may moderate the effects of other adverse characteristics on mental health outcomes (21–23), it was expected that children whose mothers had high levels of NCE (MTG 2) might have better mental health outcomes than their counterparts whose mothers had low levels of NCE (MTG 1). Interestingly, the present results indicated that the mental health outcomes of the two groups were not meaningfully different. This could reflect the fact that a child’s mental health may not be directly influenced by the mother’s neighborhood perceptions.

### Limitations

This study has several limitations of note. First, all data used is self-reported by mothers and children and may be subject to social desirability bias (42). The reduction in sample size based on the criteria that mothers and children must have been interviewed during at least three of the four time points of interest means that the sample is biased toward individuals that could be consistently reached over time. This means that the most transient families, which likely corresponds to the most underserved families (43), may not be captured in the current dataset. The family’s geographic mobility, beyond the indicator variable of whether the family had moved since the last interview wave, was also not captured in this analysis. Due to the complexity of the MTM analysis, the survey weights were not used herein, meaning that the findings are not generalizable to the full population. The unique group of mothers included in this sample, particularly the large proportion of single mothers, also hinders the generalizability of the findings to broader populations. Measures of whether the mother received mental health treatment were not incorporated into the present analysis as this information was not consistently or reliably available. Finally, though this study examines a broad period throughout the life of the mother and child, there are still large gaps in time that are not accounted for (such as between ages 9 and 15).

## Conclusion

As rates of mental disorders among adolescents continue to increase (3), it is important to better understand the constellation of factors that have a combined impact on child mental health outcomes. The present analysis allows for an understanding of how maternal mental health and neighborhood social environments change over time and ultimately impact adolescent mental health outcomes. Future work will need to identify not only the combinations of factors but also the mechanisms by which these factors impact adolescents’ mental health outcomes. Once this information is further understood, more effective interventions and support systems can be put into place. Ultimately, this will involve changes to the way clinicians approach adolescent care, by leveraging collaborations with community organizations that can aid in better understanding the family’s home environments. Furthermore, policy-level changes will be needed to help ensure that these changes to clinical practice and new collaborations are feasible. Providing appropriate mental health care to children and adolescents has the potential to greatly improve both the physical and mental health of many future generations.

## Data Availability Statement

Publicly available datasets were analyzed in this study. The data documentation can be found here: https://fragilefamilies.princeton.edu/ documentation. The data can be downloaded through the Princeton University Office of Population Research Data Archive here: https://opr.princeton.edu/archive/restricted/Default.aspx.

## Author Contributions

DL and HX developed the concept for the study. DL gathered and cleaned the data, performed all analyses, and wrote the first draft of the manuscript. HX and TL assisted with interpretation of the results. ES and DS provided subject matter expertise on neighborhood contexts and maternal-child health, respectively, to assist with contextualizing the findings. All authors reviewed and revised the original manuscript draft and approved the final version of the manuscript prior to submission.

## Conflict of Interest

The authors declare that the research was conducted in the absence of any commercial or financial relationships that could be construed as a potential conflict of interest.

## Publisher’s Note

All claims expressed in this article are solely those of the authors and do not necessarily represent those of their affiliated organizations, or those of the publisher, the editors and the reviewers. Any product that may be evaluated in this article, or claim that may be made by its manufacturer, is not guaranteed or endorsed by the publisher.

## Abbreviations

BIC: Bayesian information criteria
BSI-18: Brief Symptom Inventory 18
CES-D: Center for Epidemiologic Studies Depression Scale
CIDI-SF: Composite International Diagnostic Interview—Short Form
FFCWS: Fragile Families and Child Wellbeing Study
IQR: inter-quartile range
MD: maternal depression
MTG: multi-trajectory group
MTM: multi-trajectory modeling
NCE: neighborhood collective efficacy
SD: standard deviation
CI: confidence interval.
