# Supplementary material for: The Longitudinal Impact of Maternal Depression and Neighborhood Social Context on Adolescent Mental Health
Source: Front Pediatr. 2022 Jun 23;10:854418. doi: 10.3389/fped.2022.854418 (PMC9261195; doi:10.3389/fped.2022.854418)
Supplement: Supplementary file 1 [file Table_1.DOCX]

**Supplemental Table S1.** Listing of items for all measures of interest

| **Items** | **Response Options** |
| --- | --- |
| **Maternal Depression: CIDI-SF** |  |
| During the past 12 months, has there ever been a time when you felt sad, blue, or depressed for two or more weeks in a row? | Yes / No |
| For the next two questions, please think of the two-week period during the past 12 months when these feelings were worst. During that time, did the feelings of being sad, blue, or depressed usually last… | All day long / Most of the day / About half of the day / Less than half of the day |
| During those two weeks, did you feel this way… | Every day / Almost every day / Less often |
| During those two weeks did you lose interest in most things like hobbies, work, or activities that usually give you pleasure? | Yes / No |
| During the past 12 months, has there ever been a time lasting two weeks or more when you lost interest in most things like hobbies, work, or activities that usually give you pleasure? | Yes / No / No, on medication/anti-depressants |
| For the next few questions, please think of the two-week period during the past 12 months when you had the most complete loss of interest in things. During that two-week period, did the loss of interest usually last… | All day long / Most of the day / About half of the day / Less than half of the day |
| Did you feel this way every day, almost every day, or less often during the two weeks? | Every day / Almost every day / Less often |
| Thinking about those same two weeks, did you feel more tired out or low on energy than is usual for you? | Yes / No |
| During these two weeks, did you gain or lose weight without trying, or did you stay about the same? | Gain / Lose / Both gained and lost weight / Stay about the same / Was on a diet |
| About how much did (you gain/you lose/your weight change) during these two weeks? | Integer value in pounds |
| Did you have more trouble falling asleep than you usually do during those two weeks? | Yes / No |
| Did that happen every night, nearly every night, or less often during those two weeks? | Every night / Nearly every night / Less often |
| During those two weeks, did you have a lot more trouble concentrating than usual? | Yes / No |
| People sometimes feel down on themselves, no good, or worthless. During that two week period, did you feel this way? | Yes / No |
| Did you think a lot about death--either your own, someone else's, or death in general during those two weeks? | Yes / No |
| **Maternal Neighborhood Collective Efficacy** |  |
| *Introductory text: For each item I read, please tell me how likely it would be for your neighbors to do something or get involved…* |  |
| If children were skipping school and hanging out on the street. | Very likely / Somewhat likely / Not very likely / Very unlikely |
| If children were spray painting buildings with graffiti. | Very likely / Somewhat likely / Not very likely / Very unlikely |
| If children were showing disrespect to an adult. | Very likely / Somewhat likely / Not very likely / Very unlikely |
| If a fight broke out in front of the house or building. | Very likely / Somewhat likely / Not very likely / Very unlikely |
| If the fire station closest to the neighborhood was threatened and its budget was cut. | Very likely / Somewhat likely / Not very likely / Very unlikely |
| *Introductory text: Now I’m going to read some statements about your neighborhood and the people who live there. For each statement, please tell me if you strongly agree, somewhat agree, somewhat disagree, or strongly disagree.* |  |
| People around here are willing to help their neighbors. | Strongly agree / Somewhat agree / Somewhat disagree / Strongly disagree |
| This is a close-knit neighborhood. | Strongly agree / Somewhat agree / Somewhat disagree / Strongly disagree |
| People in this neighborhood generally don’t get along with each other. | Strongly agree / Somewhat agree / Somewhat disagree / Strongly disagree |
| People in this neighborhood do not share the same values | Strongly agree / Somewhat agree / Somewhat disagree / Strongly disagree |
| Gangs are a problem in this neighborhood | Strongly agree / Somewhat agree / Somewhat disagree / Strongly disagree |
| **Adolescent Depression: CES-D Subscale** |  |
| *Introductory text: Thinking about how you have behaved or felt during the past four weeks, please tell me whether you strongly agree, somewhat agree, somewhat disagree, or strongly disagree with the following statements.* |  |
| I feel I cannot shake off the blues, even with help from my family and my friends | Strongly agree / Somewhat agree / Somewhat disagree / Strongly disagree |
| I feel sad. | Strongly agree / Somewhat agree / Somewhat disagree / Strongly disagree |
| I feel happy. | Strongly agree / Somewhat agree / Somewhat disagree / Strongly disagree |
| I feel life is not worth living. | Strongly agree / Somewhat agree / Somewhat disagree / Strongly disagree |
| I feel depressed. | Strongly agree / Somewhat agree / Somewhat disagree / Strongly disagree |
| **Adolescent Anxiety: BSI-18 Anxiety Subscale** |  |
| *Introductory text: Thinking about how you have behaved or felt during the past four weeks, please tell me whether you strongly agree, somewhat agree, somewhat disagree, or strongly disagree with the following statements.* |  |
| I have spells of terror or panic. | Strongly agree / Somewhat agree / Somewhat disagree / Strongly disagree |
| I feel tense or keyed up. | Strongly agree / Somewhat agree / Somewhat disagree / Strongly disagree |
| I get suddenly scared for no reason. | Strongly agree / Somewhat agree / Somewhat disagree / Strongly disagree |
| I feel nervous or shaky inside. | Strongly agree / Somewhat agree / Somewhat disagree / Strongly disagree |
| I feel fearful. | Strongly agree / Somewhat agree / Somewhat disagree / Strongly disagree |
| I feel so restless I can’t sit still. | Strongly agree / Somewhat agree / Somewhat disagree / Strongly disagree |

**Supplemental Table S2.** Comparison of demographic characteristics and measures of interest among individuals included in analytic sample versus those excluded

| **Variable label** | **Characteristic** | **Not in analytic sample** | **Included in analytic sample** | **p-value** |
| --- | --- | --- | --- | --- |
| Child’s biological sex | Male | 1191 (53.41) | 1365 (51.18) | 0.120 |
|  | Female | 1039 (46.59) | 1302 (48.82) |  |
| Mother's age when the child is 3 years old |  | 28.09 +/- 6.06 | 28.28 +/- 6.07 | 0.311 |
| Mother’s race | White, non-Hispanic | 437 (19.64) | 593 (22.28) | < 0.001 |
|  | Black, non-Hispanic | 976 (43.87) | 1350 (50.73) |  |
|  | Hispanic | 714 (32.09) | 622 (23.37) |  |
|  | Other | 98 (4.4) | 96 (3.61) |  |
| Mother's education when the child is 3 years old | Less than high school | 553 (35.4) | 628 (23.56) | < 0.001 |
|  | High school or equivalent | 431 (27.59) | 772 (28.96) |  |
|  | Some college or technical school | 425 (27.21) | 888 (33.31) |  |
|  | College degree or higher | 153 (9.8) | 378 (14.18) |  |
| Mother's relationship with the father when the child is 3 years old | Married to father | 467 (29.9) | 889 (33.38) | 0.088 |
|  | Not married but living with father | 325 (20.81) | 495 (18.59) |  |
|  | Not living with father but romantically involved | 77 (4.93) | 157 (5.9) |  |
|  | Separated, divorced, or widowed | 98 (6.27) | 171 (6.42) |  |
|  | Friends with father | 289 (18.5) | 453 (17.01) |  |
|  | No relationship with father | 306 (19.59) | 498 (18.7) |  |
| Maternal cigarette use | No | 722 (73.67) | 1757 (77.71) | 0.013 |
|  | Yes | 258 (26.33) | 504 (22.29) |  |
| Maternal alcohol use | No | 800 (51.45) | 1274 (47.95) | 0.028 |
|  | Yes | 755 (48.55) | 1383 (52.05) |  |
| Maternal illicit drug use | No | 1449 (92.94) | 2468 (92.64) | 0.715 |
|  | Yes | 110 (7.06) | 196 (7.36) |  |
| Any violence exposure | No | 616 (62.03) | 1438 (63.8) | 0.337 |
|  | Yes | 377 (37.97) | 816 (36.2) |  |
| Moved homes since last interview | No | 745 (47.63) | 1422 (53.34) | < 0.001 |
|  | Yes | 819 (52.37) | 1244 (46.66) |  |
| Any material hardship | No | 883 (56.78) | 1461 (54.99) | 0.257 |
|  | Yes | 672 (43.22) | 1196 (45.01) |  |
| Household income at or below poverty level | No | 832 (53.2) | 1623 (60.85) | < 0.001 |
|  | Yes | 732 (46.8) | 1044 (39.15) |  |
| Maternal depression score at child’s age 3 |  | 1.19 +/- 2.4 | 1.21 +/- 2.42 | 0.775 |
| Any maternal depression at child’s age 3 | No | 1231 (79.06) | 2100 (78.83) | 0.858 |
|  | Yes | 326 (20.94) | 564 (21.17) |  |
| Maternal depression score at child’s age 3, among mothers with any symptomology only |  | 5.66 +/- 1.47 | 5.7 +/- 1.43 | 0.682 |
| Maternal neighborhood collective efficacy score at child’s age 3 |  | 17.13 +/- 6.31 | 17.45 +/- 6.38 | 0.195 |
| Adolescent depression score |  | 16.92 +/- 3.04 | 17.03 +/- 2.98 | 0.361 |
| Adolescent anxiety score |  | 19.1 +/- 3.95 | 19.13 +/- 3.9 | 0.876 |

**Supplemental Table S3.** Model fit statistics for all multi-trajectory models assessed

| **Number of Trajectories** | **BIC*** | **BIC Change** | **AIC**** | **AIC Change** |
| --- | --- | --- | --- | --- |
| **1** | 100785.14 |  | -50361.05 |  |
| **2** | 85573.82 | -15211.32 | -42723.88 | 7637.17 |
| **3** | 82779.78 | -2794.04 | -41365.93 | 1357.95 |
| **4** | 81016.72 | -1763.06 | -40382.28 | 983.65 |
| **5** | 79193.34 | -1823.38 | -39439.07 | 943.21 |
| **6** | 79243.74 | 50.40 | -39432.76 | 6.31 |

* BIC: Bayesian Information Criteria; **AIC: Akaike Information Criteria
